# Sediment tolerance mechanisms identified in sponges using advanced imaging techniques

**DOI:** 10.7717/peerj.3904

**Published:** 2017-11-16

**Authors:** Brian W. Strehlow, Mari-Carmen Pineda, Alan Duckworth, Gary A. Kendrick, Michael Renton, Muhammad Azmi Abdul Wahab, Nicole S. Webster, Peta L. Clode

**Affiliations:** 1 School of Biological Sciences, University of Western Australia, Crawley, WA, Australia; 2 Centre for Microscopy, Characterisation and Analysis, University of Western Australia, Crawley, WA, Australia; 3 Oceans Institute, University of Western Australia, Crawley, WA, Australia; 4 Australian Institute of Marine Science, Cape Ferguson, QLD, Australia; 5 Western Australian Marine Science Institution, Crawley, WA, Australia; 6 School of Agriculture and Environment, University of Western Australia, Crawley, WA, Australia; 7 Australian Centre for Ecogenomics, University of Queensland, Brisbane, QLD, Australia

**Keywords:** Sponge, Sediments, 3D X-ray microscopy, Scanning electron microscopy

## Abstract

Terrestrial runoff, resuspension events and dredging can affect filter-feeding sponges by elevating the concentration of suspended sediments, reducing light intensity, and smothering sponges with sediments. To investigate how sponges respond to pressures associated with increased sediment loads, the abundant and widely distributed Indo-Pacific species *Ianthella basta* was exposed to elevated suspended sediment concentrations, sediment deposition, and light attenuation for 48 h (acute exposure) and 4 weeks (chronic exposure). In order to visualise the response mechanisms, sponge tissue was examined by 3D X-ray microscopy and scanning electron microscopy (SEM). Acute exposures resulted in sediment rapidly accumulating in the aquiferous system of *I. basta*, although this sediment was fully removed within three days. Sediment removal took longer (>2 weeks) following chronic exposures, and *I. basta* also exhibited tissue regression and a smaller aquiferous system. The application of advanced imaging approaches revealed that *I. basta* employs a multilevel system for sediment rejection and elimination, containing both active and passive components. Sponges responded to sediment stress through (i) mucus production, (ii) exclusion of particles by incurrent pores, (iii) closure of oscula and pumping cessation, (iv) expulsion of particles from the aquiferous system, and (v) tissue regression to reduce the volume of the aquiferous system, thereby entering a dormant state. These mechanisms would result in tolerance and resilience to exposure to variable and high sediment loads associated with both anthropogenic impacts like dredging programs and natural pressures like flood events.

## Introduction

Sediments are an important component of all aquatic ecosystems, and they are particularly evident in coastal zones, where sediment loads and suspended sediment concentrations (SSCs) can be high and variable. High sediment concentrations in the water column can be caused by flooding or resuspension events driven by severe storms. Wave action and currents can also resuspend sediments introduced via river discharge and flooding, resulting in sediments being transported throughout nearshore marine ecosystems. Due to increased land development in coastal areas, sediment loads from riverine discharge are increasing in many coastal zones (e.g., Kroon et al., 2013). Coastal development can also have a direct impact on sediment loads through dredging activities, which involves the removal of sediment from the seafloor and generation of a sediment plume. Dredging is typically undertaken to improve access and navigation by ships, and both the number and size of dredging programs are increasing with increased port developments in Australia and globally (Ports Australia, 2014; Ports Australia, 2015). However, the potential impact of increased sediment loads from dredging and coastal runoff on marine organisms and ecosystems is understudied (reviewed in Jones et al., 2016; Jones, Ricardo & Negri, 2015; Schönberg, 2016; Fraser et al., 2017).

Sediment causes three main abiotic stressors to marine organisms: (i) elevated SSCs, (ii) increased light attenuation, and (iii) increased sediment deposition on the benthos (Jones et al., 2016). Elevated SSCs can clog gills and filter feeding apparatuses (Bell et al., 2015; Jones et al., 2016; Schönberg, 2016) and can reduce the total amount of photons and change the spectral attenuation of photosynthetically active radiation (PAR) reaching the benthos, thereby inhibiting photosynthesis (Bell et al., 2015; Jones et al., 2016; Schönberg, 2016). High sediment deposition can smother benthic organisms and impair recruitment (Jones, Ricardo & Negri, 2015).

Sponges are ecologically important benthic filter feeders (Bell, 2008; De Goeij et al., 2013; Maldonado, 2016) that are abundant (Flach et al., 1998; Diaz & Rützler, 2001; Heyward et al., 2010; Schönberg, 2016) and widely distributed (Van Soest et al., 2017) in coastal habitats where natural sediment loads can be high and dredging typically occurs (Abdul Wahab et al., 2017). Sponges feed, exchange gases, eliminate waste, and reproduce by pumping and filtering water through a complex system of internal channels and chambers known collectively as the aquiferous system (Bergquist, 1978). In the Caribbean, for example, dense populations of *Xestospongia muta* can pump the equivalent volume of the overlaying water column (at 30 m) in less than three days (McMurray, Pawlik & Finelli, 2014). Sponges can filter out between 75% and 99% of biotic and abiotic particles in the water, depending on particle size and the sponge species (Reiswig, 1971; Reiswig, 1974; Reiswig, 1975; Pile et al., 1997; Savarese et al., 1997). Considering these pumping rates and filtration efficiencies, elevated SSCs are predicted to cause sediments to enter and clog the sponge aquiferous system (Bell et al., 2015; Schönberg, 2015; Schönberg, 2016).

Some species appear well adapted to living in environments with high sediment loads, and there are at least 11 sponge genera explicitly named for their psammobiosis, i.e., living partially embedded in sediments, e.g., *Psammoclema*, *Psammocinia* (Schönberg, 2015). Others sponge species may be limited in distribution to areas with lower sediment loads, e.g., *Scopalina lophyropoda* (Maldonado, Giraud & Carmona, 2008). Possible mechanisms for, and rates of, sediment clogging of the aquiferous system are largely unknown and have only been experimentally demonstrated in two sponge species from one location (Tompkins-MacDonald & Leys, 2008). Sponges likely employ a mixture of passive and active mechanisms to reduce and prevent sediment accumulation within their aquiferous system. Passive mechanisms, such as self-cleaning surfaces, morphology, and orientation, could limit sediment accumulation inside and on top of sponges (Bell et al., 2015; Schönberg, 2015; Schönberg, 2016). Active mechanisms, including the production of mucus, expulsion of particles from the aquiferous system, pumping cessation and tissue sloughing, involve additional energy expenditure and may not be sustainable in the long term (Bell et al., 2015; Schönberg, 2015; Schönberg, 2016; Strehlow et al., 2016a). Note that the term ‘mucus’ is used in its broadest sense as sponge mucus has not been chemically characterised (Bythell & Wild, 2011). The details of these sediment exclusion mechanisms are not known for most sponge species and some have only been reported anecdotally.

**Figure 1:**
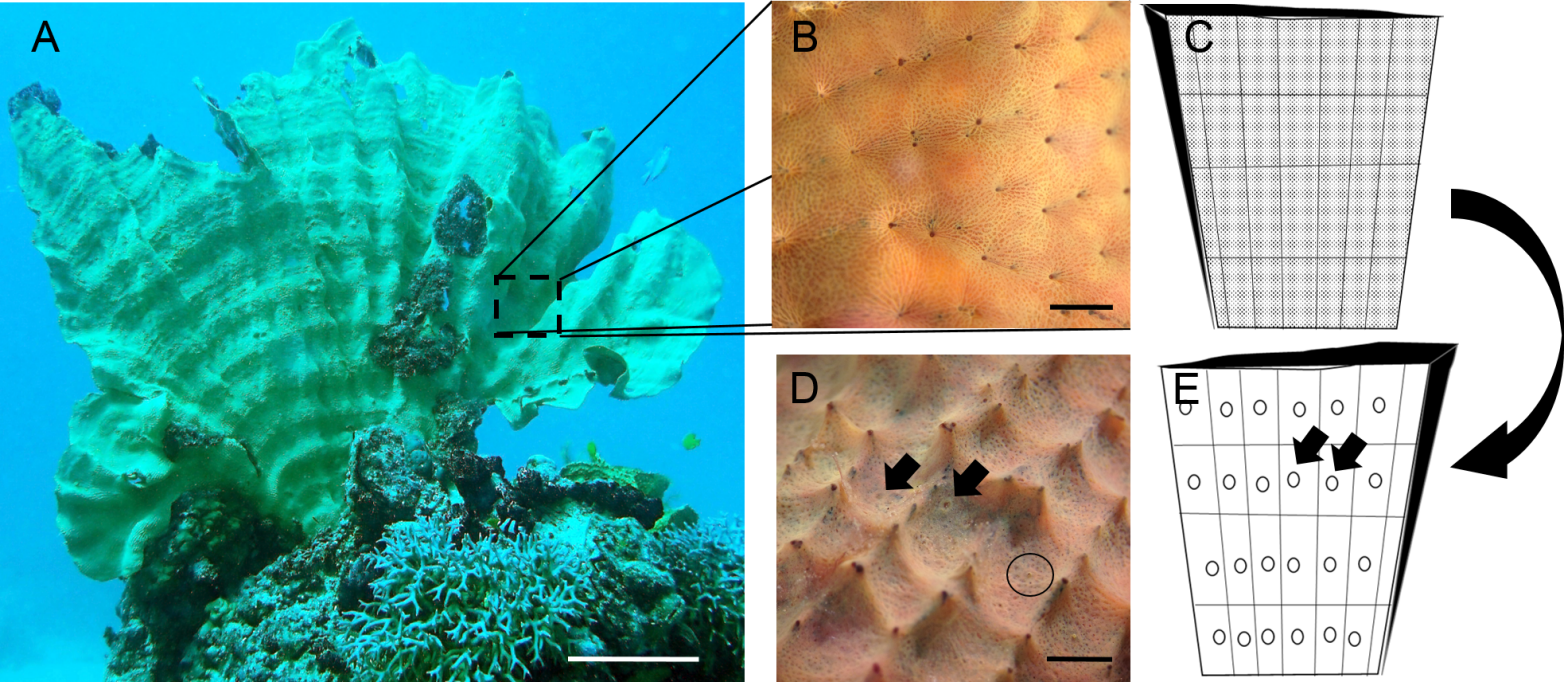
*Ianthella basta* body plan. (A) *In situ* photo of *I. basta* at Davies Reef (S 18°49.354′, E 147°38.253′). *I. basta* is generally thin (2–5 mm) and fan-shaped (scale = 5 cm). One side, the incurrent side (B), is studded with ostia (incurrent pores), and the opposite excurrent side (D), contains larger oscula (excurrent pores). (B) Close up image of incurrent surface with clusters of ostia (scale = 1 cm). (C) Schematic of incurrent surface showing many dots (ostia) and the regular structure of skeletal elements (black lines/grid). (D) Close up image of excurrent surface on the opposite side of the incurrent surface, as indicated by the large, curved arrow. Open oscula are indicated with black arrows. A contracted osculum is circled (Scale = 1 cm). (E) Schematic of excurrent surface showing many circles (oscula) and the regular structure of skeletal elements (black lines/grid). Each grid formed by the skeleton generally contains one or more contractile oscula (arrows). Photo credits: (A) Mari-Carmen Pineda; (B) and (D) Brian W. Strehlow.

To further explore the mechanistic responses of sponges to sediment we used the model species *Ianthella basta* (Pallas 1766). The widely distributed, Indo-Pacific sponge *I. basta* (Van Soest et al., 2017) is found in naturally turbid environments (Abdul Wahab et al., 2017). It is tolerant to sediment-related stress, is unaffected by light attenuation (Luter, Whalan & Webster, 2012a; Pineda et al,. 2016; Pineda et al., 2017a), has a high thermal threshold (Luter, Whalan & Webster, 2012a), and is known to recover from stress-induced tissue regression (Luter, Whalan & Webster, 2012b). It is generally thin (2–5 mm), erect and laminar (Gray, 1869; Andreakis, Luter & Webster, 2012) (Fig. 1A). Due to this thin tissue, relatively small samples (8–125 mm^3^) can be representative of the entire aquiferous system because the incurrent (ostia) and excurrent pores (oscula) are generally on opposite sides of the sponge (Figs. 1B and 1C). These features make *I. basta* an ideal candidate to investigate how sponges internally process sediments. In this study, we identified and characterised the mechanisms for internal sediment processing and tolerance in *I. basta* using high resolution three-dimensional (3D) X-ray microscopy and scanning electron microscopy (SEM).

## Materials and Methods

### Sponge and sediment collection

*I. basta* (*n* = 5, representing a single evolutionary significant unit, see Andreakis, Luter & Webster, 2012, verified via examination of skeletal structure and morphology) was collected at 10 m depth near Fantome Island (S 18°41.028′, E 146°30.706′) in the inshore, central region of the Great Barrier Reef (GBR) in Australia, leaving at least one quarter of the remaining sponge to recover (Duckworth, 2003). The number of sponges collected was limited by to five due to logistical constraints in the field. All collections from the Great Barrier Reef were performed under Great Barrier Reef Marine Park Regulations 1983 (Commonwealth) and Marine Parks regulations 2006 (Queensland) Permit G12/35236.1 and Permit G13/35758.1. Collected *I. basta* were transported in flowing seawater to the National Sea Simulator (SeaSim) at the Australian Institute of Marine Science (AIMS, Townsville), and then cut into small explants, i.e., clones, (∼1 × 3 cm) and allowed to heal for >2 weeks in large holding tanks (5,000 L) with flow-through seawater (600 mL min^−1^, 5 µm filtered) at conditions similar to the collection location (28 °C and 36‰ salinity).

Sediments were collected from two geographically diverse locations where *I. basta* is common: (i) Davies Reef, a mid-shelf reef on the central GBR (S 18°49.354′, E 147°38.253′) and (ii) a coastal habitat approximately 10 km offshore, near the township of Onslow in the Pilbara region, Western Australia (S 21°38.642′, E 114°55.924′). Sediment composition differed between collection sites, with Davies Reef sediment predominantly calcium carbonate (∼80% aragonite) and Pilbara sediment primarily siliciclastic (∼50% quartz) in nature (Ricardo et al., 2015). As such, the sediments are referred to as ‘carbonate’ or ‘siliciclastic’, respectively. Carbonate sediment were representative of sediments in offshore environments as well as sediment from specific dredging operations, including the Barrow Island in north-western Australia (see Bessell-Browne et al., 2017b). Siliciclastic sediment was representative of inshore environments as its composition was highly influenced by terrigenous discharge from a neighbouring river. The collected sediments were ground with a rod mill grinder to a mean grain size of 29 µm (range: 3–64 µm) and 18 µm (range: 2–42 µm) for carbonate and siliciclastic, respectively. Sediments were ground because fine and medium silts (<60 µm) stay in suspension longer and travel farther than other particles (Bainbridge et al., 2012), so these particles are predicted to have the largest impact in coastal areas across space and time during natural sediment resuspension events and during dredging (Jones et al., 2016). Furthermore, dredging and natural resuspension occur in both inshore and offshore environments, so it is important to understand the effects of different sediment types.

### Acute exposure to sediments

To determine if passive accumulation of internal sediments could occur without active pumping by the sponge, initial experiments were undertaken to compare sediment accumulation in living, anesthetised, and dead sponge explants. Sponges were anesthetised in a solution of 7.5% MgCl_2_ in filtered seawater (FSW) (Messenger, Nixon & Ryan, 1985) for 10 min prior to sediment exposure, resulting in closure of sponge oscula and cessation of pumping (see Strehlow et al., 2016a). To obtain dead individuals, sponges were euthanized by immersion in 100% ethanol for 10 min (Bell, 2004). Living, anesthetised, and dead explants (*n* = 3 per treatment) were exposed to elevated SSC (siliciclastic sediment, approximately 100 mg L^−1^) for 48 h. Sediments were kept in suspension using specialised 115 L tanks, as previously described (see Bessell-Browne et al., 2017a; Bessell-Browne et al., 2017b; Pineda et al., 2017b). 3D X-ray microscopy scans were assessed qualitatively for the presence of sediment (see “*X-ray microscopy settings and data quantification”*). These comparisons showed that sediment accumulated within living explants only. For dead and anesthetised explants, sediment was only present on their surfaces, validating the experimental and analytical X-ray microscopy approach and confirming that water had to be actively pumped by the sponge in order for sediment to enter the aquiferous system.

After sediment uptake by live sponges was verified, a second, acute sediment exposure experiment was performed. The experiment used the same tanks and siliciclastic sediment, and tested three SSCs: high (135 ± 30 mg L^−1^, mean ± standard error), medium (42 ± 8 mg L^−1^), and control (0 mg L^−1^), with three tank replicates per treatment, resulting in a total of nine tanks, each with five sponge explants. Sponges cloned from different ‘parent’ individuals were randomly distributed across the tanks and treatments to minimise the potential impact of genetic identity. Sediment was added every 6 h over 48 h, after which sediment addition was stopped and explants were observed for a subsequent three-week recovery period. During this recovery period, both high and medium sediment treatments returned to control levels. These SSCs correspond to levels that can be observed during dredging (Jones et al., 2015; Fisher et al., 2015) and following flooding and natural resuspension events (Bainbridge et al., 2012). Sedimentation rates, i.e., the rate at which sediments deposited on the bottom of the tank, was measured for each SSC treatment using SedPods (Field, Chezar & Storlazzi, 2013). Sedimentation rates were 1.29 ± 0.07, 1.03 ± 0.05, and 0.14 ± 0.01 mg cm^−2^ d^−1^ in the high medium and control, respectively. The deposition detected in the control treatment was comparatively low, and was likely caused by algal growth or the particulate waste produced by the sponges, i.e., not sediments. The natural orientation, i.e., erect and perpendicular to the substratum, of *I. basta* was preserved during all experiments. Therefore sediment deposition on the sponge surface was not equal to the sedimentation rates on the SedPods, and the effect of sedimentation on the sponges was presumed to be minimal. Daily light integrals were approximately 1.2, 3.0, and 6.5 mol photons m^−2^ d^−1^, respectively. However, as *I. basta* is a heterotroph, decreased light levels have no effect its physiology (Pineda et al., 2016). Therefore, due to its orientation and heterotrophy, the potentially confounding effects of light attenuation and sediment smothering were eliminated in *I. basta*.

Sponges were sampled 24 h after sediment dosing commenced and after 1 h, 24 h, 78 h, and 3 weeks (w) of recovery under control conditions and fixed for X-ray microscopy and SEM analysis. Three separate sponges were processed per time point, one from each tank. Oscula on each sponge at each sampling time, as well as after 3 h of recovery, were recorded as either open or closed where visible. Sponge explants contained between 3–10 oscula.

### Chronic exposure to sediments

In the longer-term (chronic) exposure experiment, large (∼5 × 5 cm) *I. basta* explants were exposed to elevated SSCs (76 mg L^−1^), reduced light (daily light integral: 0.15 mol photons m^−2^ d^−1^), and high sedimentation rates (5.2 mg cm^−2^ d^−1^) for four weeks using the carbonate sediment. In the control treatment, sponges were exposed to no suspended sediments, no sedimentation, and a higher daily light integral of 6.53 mol photons m^−2^ d^−1^, typical levels for clear water reef environment (Jones et al., 2015; Fisher et al., 2015). Experiments were performed in 4 × 1,200 L fibreglass tanks with 5 µm filtered seawater in an environmentally controlled room at the SeaSim. The chronic exposure formed part of a larger collection of studies (Pineda, Duckworth & Webster, 2016; Pineda et al., 2017a; Pineda et al., 2017b; Pineda et al., 2017c) using carbonate sediment. Sediment was kept in suspension using two recirculating pumps (see Pineda et al., 2017a). When sediment concentrations dropped, doses of concentrated sediment (∼6 g L^−1^) were added automatically. The doses were monitored and controlled throughout the experiment by integrated nephelometers, which measured sediment levels, connected to a programmable logic controller (PLC) (see Pineda et al., 2017a). Two tank replicates were used for each treatment, making four tanks in total. At each time point, one sponge was taken from one tank and two sponges were taken from the corresponding tank replicate, making three replicates per time point per treatment and a total of 12 sponges. A piece (∼1 × 3 cm) was fixed for X-ray microscopy, as outlined below, after four weeks of exposure to both control and high sediment conditions from each replicate (*n* = 6). After four weeks, tanks with high sediment loads were returned to control conditions to allow sponges to recover. After 2 w of recovery, a piece (∼1 × 3 cm) was taken from each replicate (*n* = 6) as described above and fixed for examination using X-ray microscopy. For a full account of the methods and specifications of this experiment see: Pineda et al. (2017b).

### X-ray microscopy settings and data quantification

Sponges were fixed in 2.5% glutaraldehyde and 1% paraformaldehyde in FSW for 1 h at 23 °C and stored at 4 °C. Sponges were stained in diluted Lugol’s Iodine (I_2_: 1%, KI: 2%) for 24 h, to increase the contrast of tissue elements in X-ray scans. Three-dimensional X-ray scans were performed using an Xradia Versa XRM520 X-ray microscope, using the following specifications: ∼5 µm pixel size; voltage: 80 kV; power: 7 W; exposure time: 12 s; and binning: 2. Scanning time was approximately 6 h per sample. Projections were reconstructed using XMReconstructor software (Xradia Inc., Pleasanton, CA, USA).

Avizo Fire software (FEI, Hillsboro, OR, USA) was used to extract quantitative data from the 3D reconstructions. The Edit New Label Field tool was used to segment scans. Due to the porous nature of sponges, the sponge tissue could not be segmented out from the background using thresholding. Instead, the lasso selection tool was used to manually select the internal edge of the pinacoderm (outer layer) on 2D *X*–*Y* cross sections. Selections were performed every 50 cross-sections (∼250 µm), and then the interpolate function was used to create a 3D selection between the two selections. This process was repeated across 800 cross-sections (∼4 mm) to select the internal volume of the sponge. This procedure also served to exclude sediments on the surface of the sponge since the concentration of these sediments was highly variable due to handling effects and mucus production (Pineda et al., 2017a). Internal organisms, such as polychaetes and barnacles, were manually excluded from analysis using the lasso selection tool. The internal sediment, within the selected volume, was then segmented via the Interactive Thresholding tool, and the number of sediment particles and sediment particle volumes was quantified using the Label Analysis tool. Sediment particle diameter, was estimated assuming that the particles were spherical. Sponge tissue and skeletal elements were also segmented, labelled, and quantified within the selected volume. The remaining volume in the selection was labelled as aquiferous system because it matched threshold levels of the background solution, and was quantified using the same approach. The number of sediment particles in each sample was standardised to the selected volume, i.e., aquiferous system volume plus tissue and skeletal volumes. During tissue regression in *I. basta*, the choanocyte chamber density decreases and the tissue itself appears denser (Luter, Whalan & Webster, 2012b), indicating that the volume of the aquiferous system channels had decreased. The potential for rapid recovery from this regressed state implies that there is little change in the total number of sponge cells present (Luter, Whalan & Webster, 2012b). Therefore, in order to quantify tissue regression, the percent of the total volume occupied by the aquiferous system was calculated. Due to the pixel size, the aquiferous system volume analysed is more representative of the channels of the aquiferous system, i.e., incurrent channels, excurrent channels, atrium, etc., than of the choanocyte chambers, which are difficult to visualise at this resolution and within these relatively large samples.

Sediment particle position was determined by examining cross sections of the 3D volumes. A random cross section was selected for each sample. If sediment particles appeared tightly surrounded by sponge tissue, they were considered to be in the mesohyl. Particles present in the channels were also counted. The percentage of total particles in the mesohyl and in the channels was determined for each sample and averaged for each collection point to determine sediment position.

### SEM analysis

Fixed sponges were dehydrated in increasing concentrations of ethanol using a PELCO Biowave microwave fitted with a PELCO Coldspot, then immersed in liquid nitrogen and fractured using a chisel (Johnston & Hildemann, 1982). This fracturing technique revealed the full aquiferous system including the choanocyte chambers. After fracturing, samples were returned to anhydrous ethanol, critical-point dried, and then mounted on stubs using carbon tape. Mounted samples were coated with 10 nm of gold and 10 nm of carbon to ensure sample conductivity. Images were acquired at 5 kV using a Zeiss 55 field emission scanning electron microscope. After 24 h exposure, all samples from the acute exposure experiment were assessed qualitatively for the presence of sediment. One control sample from each time point was also mounted without being freeze fractured in order to view the outer surface. Ostia, incurrent pore diameter, and surface area was determined from images using the ImageJ software package (Rasband, 2012).

**Table 1:** AIC values for models generated for the different response variables for the acute and chronic exposures to sediment. The chosen model based on the lowest AIC score is shown in bold.

Response variable	Acute exposure to siliciclastic sediment				Chronic exposure to carbonate sediment			
	ANOVA with Tank	ANOVA without tank	GLS with tank	GLS without tank	ANOVA with Tank	ANOVA without tank	GLS with tank	GLS without tank
Sediment particles mm^−3^	403	367	362	**360**	86	77	84	**75**
Sediment particle volume	272	**270**	N/A	N/A	63	**61**	N/A	N/A
Percent volume aquiferous system	375	**354**	N/A	N/A	−16	**−18**	N/A	N/A

### Statistical analyses

Linear models were fitted to predict internal sediment variables (i.e., sediment particles mm^−3^ and sediment particle volume) and the percent volume of the sponge aquiferous system in terms of time and treatment (both as fixed factors). Control sponges had no internal sediment at any time, so control treatments from internal sediment variables data were excluded from the models. Internal sediment data were then compared between sediment treatments and over time. The percent volume of the sponge aquiferous system from control treatments was included in the models. These models were compared to linear mixed effects models with an additional random factor for tank, using the Akaike information criterion (AIC) in R (R Development Core Team, 2016). Based on the lowest AIC values, there was no indication that tank had an effect on any variable in either experiment, so the more parsimonious linear models, i.e., without tank as a random factor, were used (Table 1). In order to minimise genetic effects, sponge explants from each original replicate were randomly distributed between the tanks. However, due to the low number of original replicates (*n* = 5), there was still a chance of false positives in the models due the limited genetic independence of the explants. For models of sediment particles mm^−3^ over time and treatment, variances were weighted using a power law function within a generalised least squares (GLS) model for data from both acute and chronic exposure, in order to ensure homogenous variance of standardised residuals. Power-law weighted GLS models were the best fit according to AIC value for sediment particles mm^−3^ in both exposures. All other variables were modelled using linear models, i.e., analyses of variance (ANOVA). Data were arcsine transformed to model the percent volume of aquiferous system relative to tissue and skeletal volume over time and treatment in the chronic exposure, to meet the assumption of a normal distribution; otherwise raw data were used. Significance of model terms was evaluated using *F*-tests following the standard ANOVA approach. Tukey HSD was used to test for differences between time points and treatments. Welch *t*-tests were performed for each time point to identify differences in internal sediment mm^−3^ in each treatment level, i.e., medium and high sediment, in the acute exposure. Unless otherwise specified, reported values represent mean plus or minus standard error.

## Results

### Acute exposure to sediments

**Figure 2:**
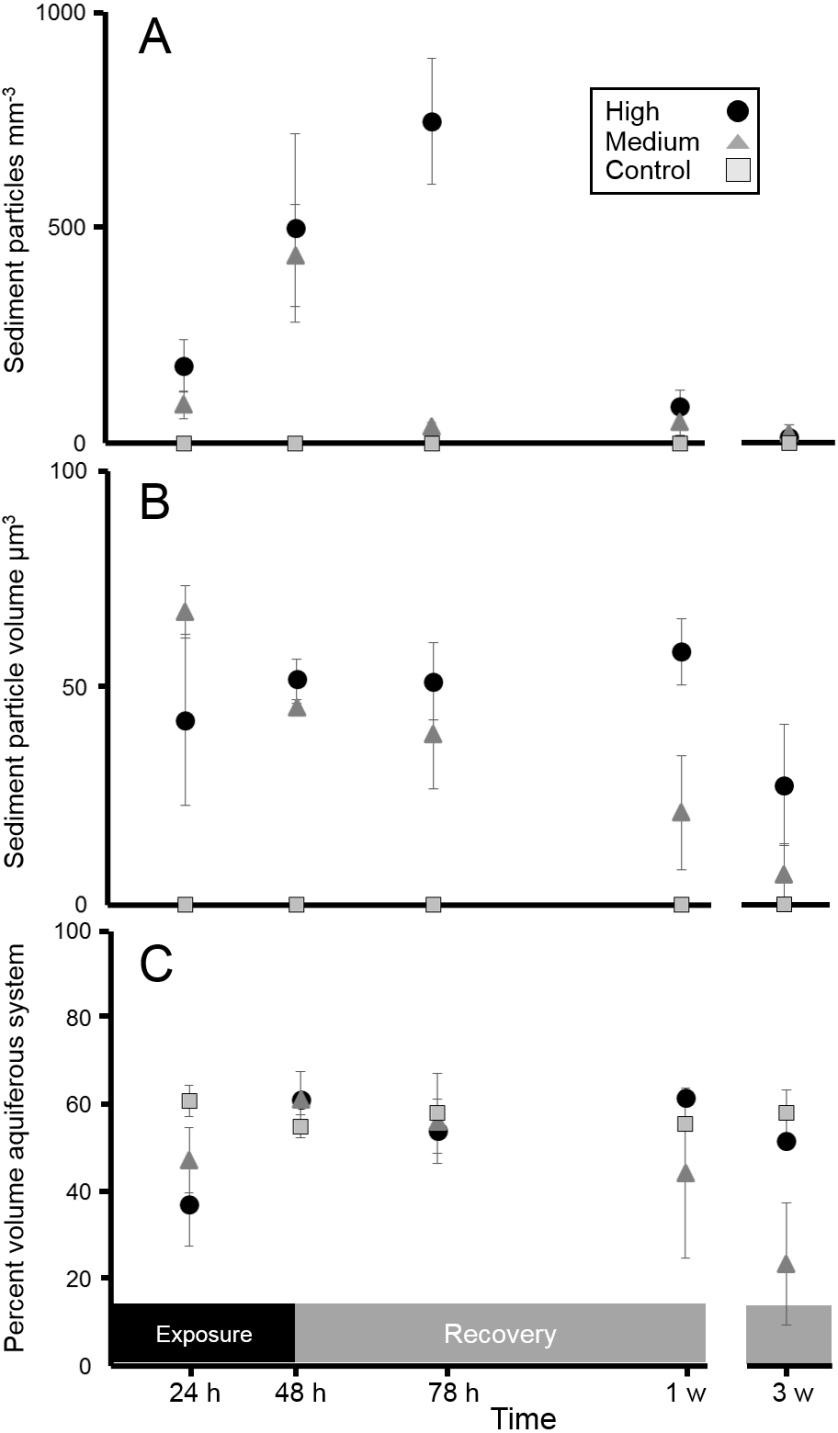
Siliciclastic sediment inside *I. basta* during the acute 48 h exposure experiment and subsequent recovery. Data (mean ± SE) show (A) the number of internal sediment particles mm^3^, (B) sediment particle volume (µm^3^), and (C) volume of aquiferous system relative to tissue and skeletal volume (%), during the exposure and recovery periods (black and grey bars, respectively) for the control (0 mg L^−1^, grey squares), medium (42 ± 8 mg L^−1^, dark grey triangles), and high sediment (135 ± 30 mg L^−1^, black circles) treatments.

**Figure 3:**
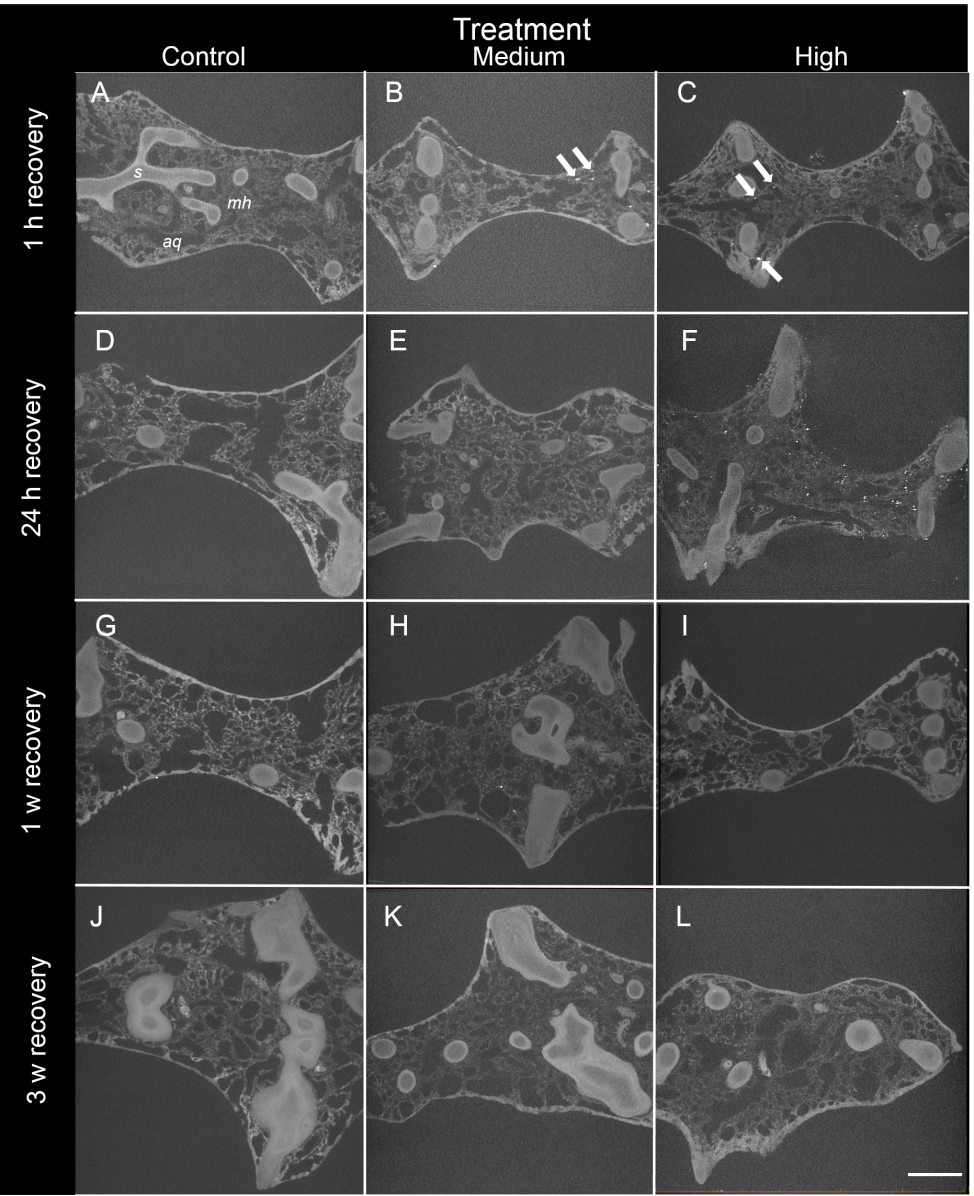
Representative 2D X-ray cross-sections of *I. basta* during the recovery period after 1 h, 24 h, 1 w, and 3 w for the control (0 mg L^−1^), medium (42 ± 8 mg L^−1^), and high siliciclastic sediment (135 ± 30 mg L^−1^) treatments. The sponge skeletal fibres (*s*), mesohyl (*mh*) and aquiferous system channels (*aq*) are visible in all treatments. As recovery time increases, the sediment (white particles indicated by arrows) becomes less concentrated, and no sediment was observed in the control sponges (scale = 1 mm).

**Figure 4:**
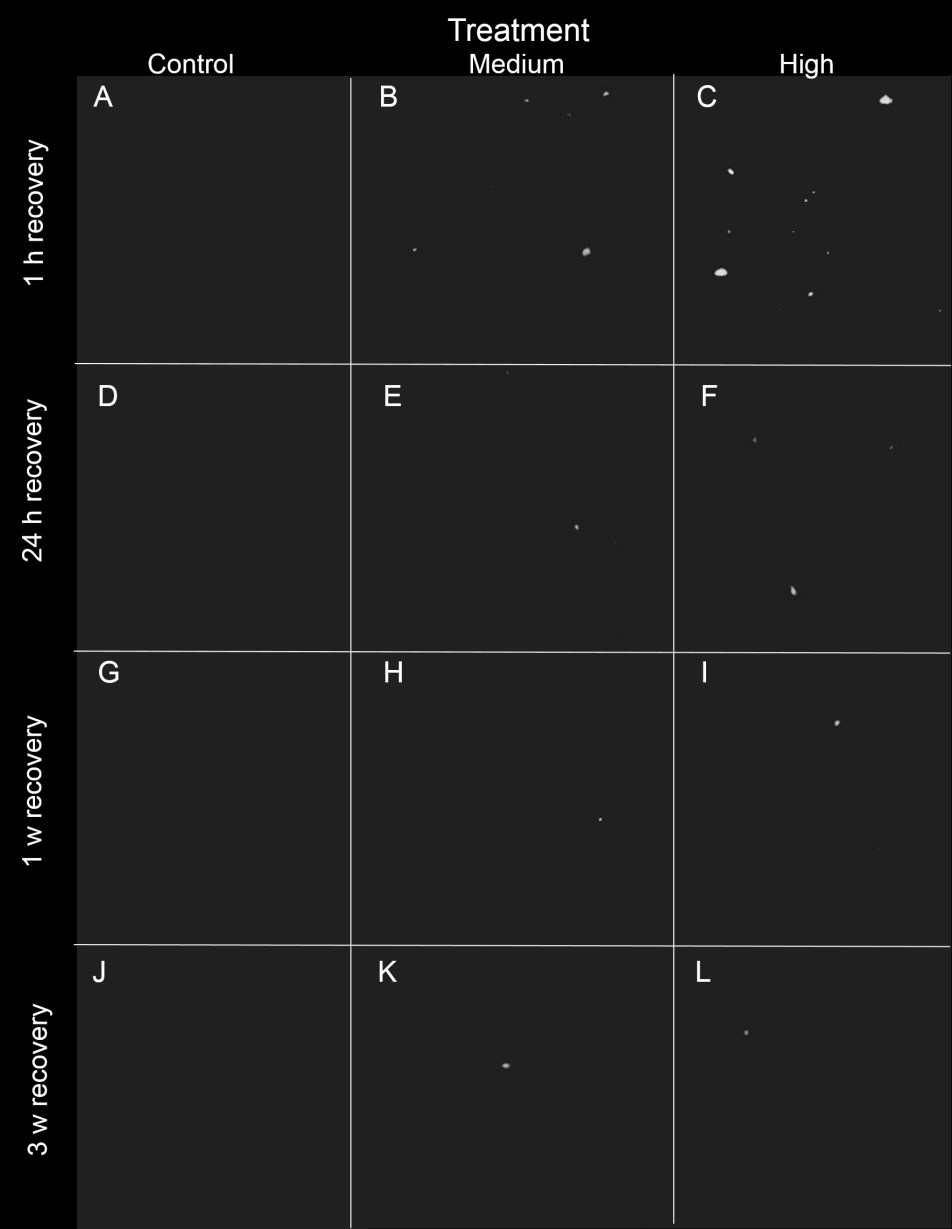
Representative 3D X-ray images of siliciclastic sediments within *I. basta* during the recovery period after 1 h, 24 h, 1 w, and 3 w for the control (0 mg L^−1^), medium (42 ± 8 mg L^−1^), and high (135 ± 30 mg L^−1^) sediment treatments. Sponge tissue (removed via thresholding) is not shown in order to emphasise sediment particles and distribution. As recovery time increases, the sediment (white) becomes less concentrated, and no sediment was observed in the control sponges (no scale is shown because the particles are distributed in 3D).

After 24 h of exposure, sediment was present in *I. basta* from both high and medium SSC treatments, but was not evident in control sponges (Figs. 2–4). No sediment particles were evident in the control sponges at any time point (Figs. 2–4). Internal sediment particles per cubic millimetre of the total volume, i.e., of the sponge tissue and the aquiferous system, (sediment particles mm^−3^) increased significantly following exposure and then decreased during the recovery (*P* < 0.01, Table 2A), and there was a significant interaction of treatment (i.e., sediment level) with time (*P* < 0.05, Table 2A). After the first hour of exposure, the number of sediment particles in the high treatment sponges (180 ± 61 particles mm^−3^) was double that of the medium treatment (90 ± 32 particles mm^−3^), however this was not significantly different (*P* > 0.05). After 48 h of exposure and 1 h of recovery, sponges in the high and medium treatments had accumulated 500 ± 218 and 435 ± 118 particles mm^−3^ respectively, and again there was no significant difference between the two sediment treatments (*P* > 0.05, Table 2A, Fig. 2). Two dimensional (2D) cross-sections, of 3D X-ray microscopy images for all treatments are shown in Fig. 3. These cross-sections show the sponge skeletal and aquiferous system elements as well as the mesohyl and any internal sediments. Representative 3D X-ray microscopy images, thresholded to show only internal sediments are shown in Fig. 4. Full 3D renderings of sponges and sediments are presented in Movies S1–S5 .

After ∼24 h recovery (78 h after initial sediment exposure), sediment levels inside samples from the high treatment (747 ± 146 particles mm^−3^) remained significantly higher than those of the medium treatment (39 ± 10 particles mm^−3^, Welch two sample *t*-test *P* < 0.05, Table 2A Fig. 2). After 3 w of recovery, little to no sediment was detected in either of the sediment treatments or the controls (13 ± 8, 21 ± 21, and 0 ± 0 particles mm^−3^ for the high, medium, and control treatments respectively).

**Table 2:** ANOVA summaries for the acute exposure to siliciclastic sediments. (A) A generalised least squares (GLS) model with power law weighted variance of sediment particles mm^−3^ in the tissue over time and sediment treatment level. Welch *t*-test results are shown if significant. Time point 3, i.e., ∼24 h recovery (78 h after initial sediment exposure), is abbreviated as T3, and the high and medium sediment treatments are abbreviated as H and M, respectively. (B) ANOVA of sediment particle volume over time and sediment treatment level. Data from control sponges were excluded from the analysis in (A) and (B) because there were no sediments present and the values for the controls were all zero. (C) ANOVA of the arcsine transformed percent volume of the aquiferous system based on time and treatment. Control data was included in (C).

	*df*	*F*	*P*
(A) Sediment particles mm^−3^			
Treatment	1	0.59	0.45
Time	4	7.30	<0.001
Treatment: time	4	3.627	0.02
Residuals	20		
Welch *t*-test			
Treatment: time	M:T3 < H:T3; *t* = 4.84, *df* = 2.02, *P* = 0.04		
(B) Sediment particle volume			
Treatment	1	2.30	0.15
Time	4	3.63	0.02
Treatment: time	4	2.25	0.01
Residuals	20		
(C) Percent volume aquiferous system			
Treatment	2	0.19	0.83
Time	4	0.50	0.73
Treatment: time	8	1.31	0.27

All sediment exposed sponges (*n* = 3 per time point) closed all of their oscula within the first 24 h of sediment dosing in the high and medium treatments, but re-opened them at differing times. In the medium treatment, oscula reopened 1 h after sediment exposure ended, i.e., 49 h after the start of the experiment. Whereas, in the high treatment, oscula stayed closed for 3 h after sediment exposure ended. Control sponges did not exhibit closed oscula at any of the sampling times.

The size of sediments (volume: 41 ± 10 µm^3^, diameter: 4.2 ± 0.8 µm) inside the sponge changed very little over time for either sediment treatment. Although there was a significant effect of time on sediment size (*P* < 0.05 Table 2B), this effect was likely caused by the decrease in particle volume in the medium treatment after 3 w (Fig. 2B). However this decrease was not significant in the Welch *t*-test (*P* > 0.05), and no other post hoc comparisons were significantly different. The percentage of sponge aquiferous volume relative to tissue and skeletal volume similarly did not significantly decrease (*P* > 0.05, Table 2C) in any treatment, remaining at ∼56% throughout the experiment and recovery period for all treatments (Fig. 2C).

**Figure 5:**
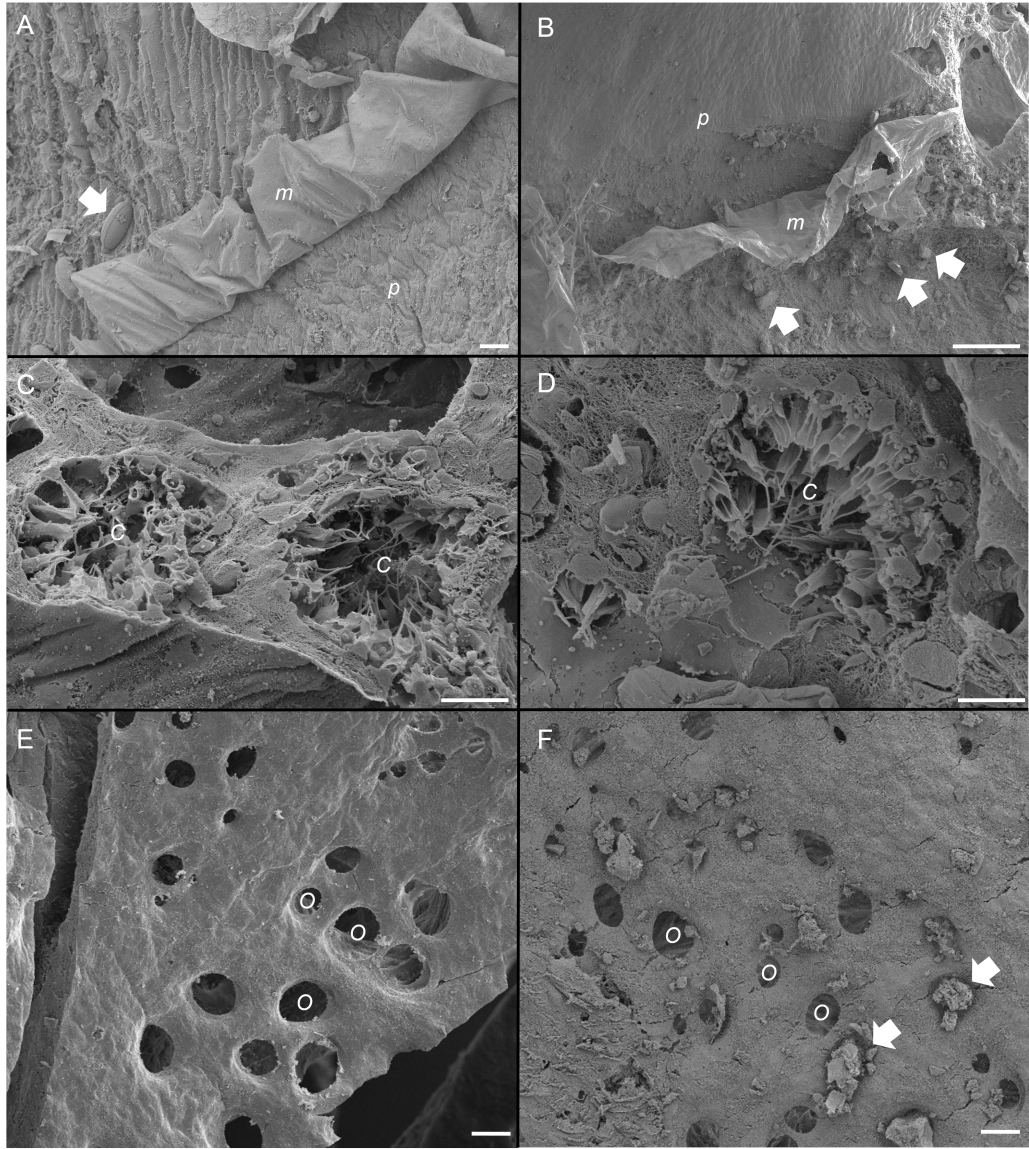
Scanning electron micrographs of the sponge, *Ianthella basta* in control (0 mg L^−1^) (A, C, E) and acute siliciclastic sediment exposure (B, D, F). (A) Mucus (*m*) production in a control sponge, trapping a diatom (arrow). Mucus sloughing cleaned an area of pinacoderm (*p*) (Scale = 10 µm). (B) Mucus (*m*) production in *I. basta* from the high sediment (135 ± 30 mg L^−1^) treatment after 48 h exposure and 1 h recovery. Sediments (arrows) were trapped in mucus (*m*). Mucus sloughing cleaned sediments off an area of pinacoderm (*p*) (Scale = 10 µm). (C) Choanocyte chambers (*C*) in the control treatment after 24 h (Scale = 10 µm). (D) Choanocyte chamber (*C*) from the high sediment treatment after 24 h of exposure to sediments (Scale = 10 µm). (E) Surface and ostia (*O*, incurrent pores) from a control after 24 h of exposure to control conditions (Scale = 20 µm). (F) Surface and ostia (*O*) from the medium sediment treatment (42 ± 8 mg L^−1^) after 24 h of exposure to sediment (Scale = 20 µm, arrows indicate sediment particles blocking ostia).

In the high sediment treatment, internal sediment was mostly located (93%) in the mesohyl of sponges after 24 h. After 48 h of exposure and during recovery, most internal sediment (61–93%) was found in the channels of the aquiferous system. In the medium sediment treatment, the majority (>65%) of internal sediments were located in the channels of the aquiferous system at all time points.

SEM revealed that all sponges, including those not exposed to sediments, produced considerable amounts of mucus on their outer surfaces (Figs. 5A and 5B). Rates of mucus sloughing and production were not determined in this study. Mucus in the control treatment appeared to trap and facilitate any fouling on the sponge surface or pinacoderm (Fig. 5A). The mucus in the siliciclastic sediment treated sponges also appeared to trap and facilitate the removal of sediment particles on the sponge’s outer surface (Fig. 5B). For the siliciclastic sediment, no major differences were observed in choanocytes of sediment-treated and control sponges (Figs. 5C and 5D). In sediment-treated and control sponges, choanocyte chambers were present and not clogged with sediments. Some sediment-like particles were observed in the channels of the aquiferous system and choanocytes, but internal sediment levels were not quantifiable. The ostia of some sponges exposed to sediment were clogged with sediment particles (Fig. 5F), whereas the ostia of control sponges were always clear (Fig. 5E). In general, ostia (*n* = 15) in control sponges were found to be ellipsoid with an average diameter of 20 ± 1 µm and an average area of 268 ± 34 µm^3^.

### Chronic exposure to sediments

**Figure 6:**
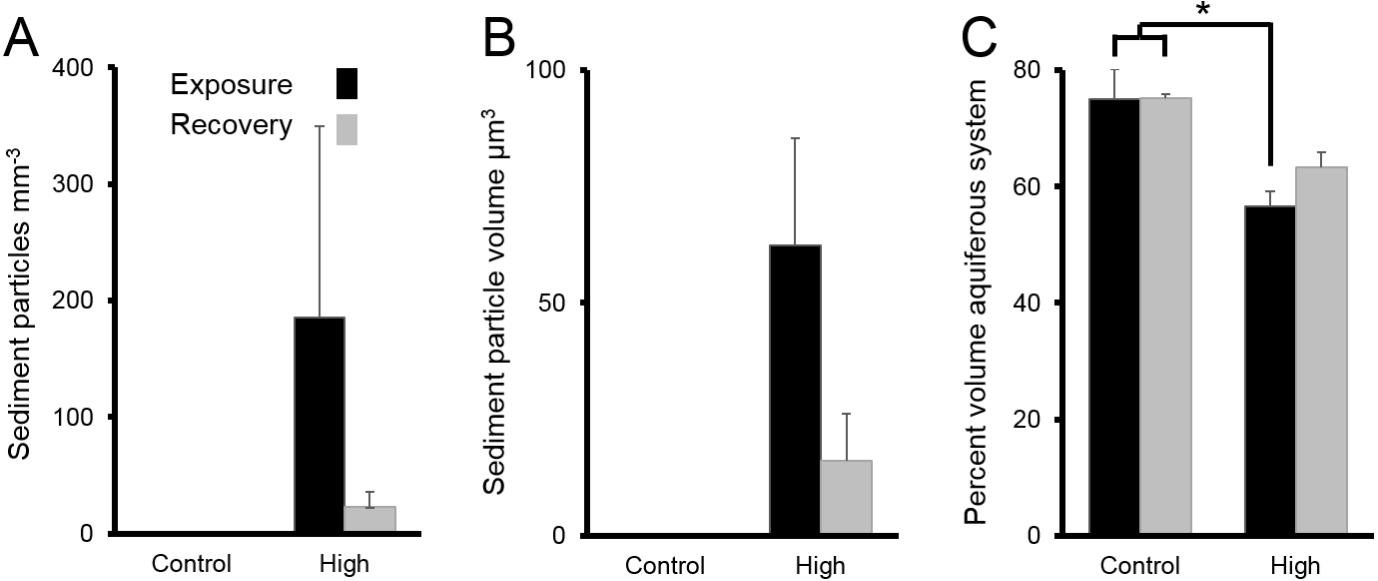
Sediments inside *Ianthella basta* after 4 w exposure (black bars) to carbonate sediments (High; 76 mg L^−1^) and control conditions (Control; 0 mg L^−1^), as well as after 2 w recovery in control conditions (grey bars). Data (mean ± SE) show the number of (A) internal sediment particles mm^−3^, (B) internal sediment particle volume (µm^3^), and (C) volume of aquiferous system relative to tissue and skeletal volume (%). Statistically significant differences are noted with an asterisk.

Following chronic exposure to carbonate sediments, no sediment was detected in control sponges. There was accumulation of sediment, with high internal sediment concentrations of 185 ± 165 particles mm^−3^, in the treated sponges after 4 w of exposure to elevated suspended sediments, light attenuation, and increased sedimentation. Internal sediment concentrations decreased to a very low level of 23 ± 13 particles mm^−3^ after 2 w of recovery in control conditions (Fig. 6A); however, this reduction was not statistically significant (*P* > 0.05, Table 3A).

**Table 3:** ANOVA summaries for chronic exposure to carbonate sediments. (A) A generalised least squares (GLS) model with power law weighted variance of sediment particles mm^−3^ in the tissue over time, i.e., 4 w exposure or 2 w recovery, in the high sediment (76 mg L^−1^) treatment. (B) ANOVA of sediment particle volume over time high sediment treatment. Data from control sponges were excluded from the analysis in (A) and (B) because there were no sediments present and the values for the controls were all zero. (C) ANOVA of the arcsine transformed percent volume of the aquiferous system based on time and treatment. Control data was included in (C). A Tukey HSD test was performed to determine differences between the high sediment treatment and the control sponges, abbreviated H and C, respectively.

	*df*	*F*	*P*
(A) Sediment particles mm^−3^			
Time	1	0.96	0.38
Residuals	4		
(B) Sediment particle volume			
Time	1	3.42	0.14
Residuals	4		
(C) Percent volume aquiferous system			
Treatment	1	12.9	<0.01
Time	1	0.30	0.60
Treatment: time	1	0.27	0.62
Residuals	8		
Tukey HSD			
Treatment	*H* > *C*, *P* < 0.01		

The average amount of sediment particles mm^−3^ in sponges from the chronic treatments after 4 w of sediment treatment (185 ± 165 particles mm^−3^) was approximately 63% lower than the highest observed value in the acute treatments, despite the longer exposure time. The average particle volume of internal sediments in the chronic exposure was 62 ± 23 µm^3^ (diameter: 5.0 ± 1.3; Fig. 6B), and it did not significantly change over time (*P* > 0.05, Table 3B).

The percent volume of the aquiferous system (relative to the tissue and skeletal volumes) in the high sediment treatment (56 ± 3%) was significantly lower after 4 w exposures than that of control treatments (75 ± 5%) (Tukey HSD: *P* < 0.01, Table 3C Fig. 6C). The percent volume of the aquiferous system did not fully recover to control levels by the end of the recovery period, returning to 63 ± 3% (*P* = 0.075). After 4 w of chronic exposure, the majority of internal sediments appeared within channels (66 ± 33%). After the 2 w recovery, the majority of internal sediments appeared in the mesohyl (75 ± 20%).

## Discussion

The sponge *Ianthella basta* was tolerant of both short term acute and longer term chronic increases in suspended sediments. *I. basta* was able to survive using a combination of passive and active strategies, specifically through the production of mucus to trap and remove surface sediment, exclude large particles from its aquiferous system via its ostia, close its oscula to limit pumping, eliminate any particles that did enter the aquiferous system, and enter into a regressed state wherein the size of the aquiferous system was reduced. These mechanisms have been reported or hypothesised in other sponge species (Bell et al., 2015; Schönberg, 2015; Schönberg, 2016), and this study extends our knowledge by providing a comprehensive account of these mechanisms occurring concurrently within a single species. This suite of mechanisms likely underpins the ecological tolerance and resilience exhibited by *I. basta* to sediment pressures (Luter, Whalan & Webster, 2012b; Pineda et al., 2017a; Pineda et al., 2017b).

Previous analyses by Pineda et al. (2017a) revealed that mucus cover on the surface of *I. basta* significantly increased from nearly zero to 21 ± 9% in the highest sediment treatment, in the chronic exposure, compared to pre-exposure and control sponges. Mucus was present on both the incurrent and excurrent side of the sponge. *I. basta* mucuslikely functions as an anti-fouling mechanism as seen in corals (Erftemeijer et al., 2012; Jones et al., 2016; Bessell-Browne et al., 2017a) and as proposed in the sponges *Carteriospongia foliascens* (Pineda et al., 2017a) and *Lamellodysidea herbacea* (Biggerstaff et al., 2017). In *I. basta*, mucus likely functioned to remove sediments in the treated sponges and remove surface fouling organisms such as diatoms in untreated sponges. The chemical composition and metabolic cost of this mucus production remains to be determined. However, based on the increased respiration rates in *L. herbacea* following chronic sediment exposure (Biggerstaff et al., 2017) and the high energetic cost of mucus production in corals (e.g., Riegl & Branch, 1995), the increased mucus production in *I. basta* may also be energetically costly. It also remains unclear how sponges slough or remove mucus. Corals use ciliated surfaces to remove mucus sheets (Stafford-Smith & Ormond, 1992), but sponges do not have ciliated outer surfaces (Bergquist, 1978). High ambient currents, such as those created in this study and in Pineda et al. (2017a), Pineda et al. (2017b) and Pineda et al. (2017c), could facilitate the removal of mucus and the particles trapped therein; however, *L. herbacea* was still able to slough mucus and trapped sediments in low-flow conditions (Biggerstaff et al., 2017). The dynamics of sponge mucus production warrant future research, for which *I. basta* would be an excellent model species.

The ostia of *I. basta* also form a passive, physical barrier against the entrance of foreign particles larger than ∼20 µm in diameter. Sponges in the acute exposure had higher internal sediment concentrations than those in the chronic treatments, which may reflect the different sediment types and sizes used in the acute and chronic experiments. The smaller particles in the siliciclastic sediment used in the acute exposure likely entered the aquiferous system more easily. However, the particle size of the internal sediments in both experiments was similar, indicating that the ostia formed a size-selection barrier for larger sediments. With the ostia acting as a sieve, the majority of internal particles had a diameter of 4–5 µm, which corresponds to very fine silts and clay. In dredge and flood plumes, sediment is typically fine and comprised of medium silts and clays (<60 µm; Bainbridge et al., 2012; Jones et al., 2016). In addition to introducing smaller sediments to the aquiferous system, dredge and flood plumes could also potentially clog ostia of *I. basta* with larger sediment particles*.* This clogging of ostia has been previously hypothesised to negatively impact sponges (Bell et al., 2015; Schönberg, 2015; Schönberg, 2016), although we now provide experimental evidence that this occurs under sediment exposure. Sponge ostia can range from 1–50 µm in diameter across taxa (Simpson, 1984). Species with larger ostia and higher choanocyte chamber density are likely to be more vulnerable to high sediment exposure, since they also have comparatively high particle clearance rates (Turon, Galera & Uriz, 1997), meaning that they can filter out more particles, including sediments. For example, the sponge *Rhopaloeides odorabile* would be susceptible by this criteria, and this vulnerability may partially explain its reduced abundance in inner-shelf reefs of the GBR, where fine-grained sediment loads are high (Bannister, Battershill & De Nys, 2012). While some sponge species can contract their ostia (reviewed in Leys & Meech, 2006; Elliott & Leys, 2007), this was not observed in *I. basta*. However, the possibility of ostia contraction in *I. basta*, particularly during coordinated tissue regression (see below), also warrants further investigation.

In *I. basta*, sediments that are not trapped in mucus or excluded by ostia can still potentially enter the aquiferous system. Once inside, they could reduce pumping efficiency and affect food uptake and gas exchange. While many studies have examined the rate at which sponges can remove particles from water, few have determined the rates at which these particles are expelled. Strehlow et al. (2016b) demonstrated that introduced *Symbiodinium* (∼10 µm in diameter) could persist in the tissues and aquiferous system of *Amphimedon erina* for five days after inoculation, which was comparable to the 3 day persistence of sediment in *I. basta* from the high sediment treatment. The majority of sediments were observed in the channels of the aquiferous system as opposed to the sponge mesohyl. The increase in mesohyl sediments in the chronic exposure following the recovery period may have occurred when sediments on the surface of the sponge were engulfed as the sponge tissues expanded to recover from regression.

Due to the high filtration efficiencies of sponges, it is likely that the sediments were taken up by the choanocytes and then expelled via exocytosis into the exhalant system. Furthermore, no clumping sediments, and no corresponding increase in sediment particle volume, were observed within the scans, which would have been expected if sediments were clogging the aquiferous system elements. This indicated efficient processing of sediments. Additionally, the lack of significant changes in the aquiferous system volume in the acute exposure indicated that a coordinated contraction of aquiferous system elements to remove clumps of material, i.e., ‘sneezing’ (Elliott & Leys, 2007; Ludeman et al., 2014), may not occur in *I. basta*. However, increasing the temporal resolution of the scans could yield further insights into this coordinated behaviour in future studies. Targeted increases in spatial resolution could also be used to better identify the various elements of the aquiferous system in the future. It may also be possible that internal sediments pass directly through the aquiferous system, without ingestion, and out through the oscula. Prosopyles and apopyles, i.e., the openings leading into and out of choanocyte chambers, respectively, were 7–8 µm in diameter (B Strehlow, pers. obs., 2017), suggesting that the small internal particles, i.e., fine silts and clays, could pass directly through the smallest part of the sponge filtration system. Sediment may also have egressed through canals that bypass choanocytes to prevent clogging (Bavestrello, Burlando & Sara, 1988; Hammel et al., 2012). Although no direct evidence of sediment ingestion, i.e., phagocytosis or intracellular sediments, was observed in these experiments, it is possible that sediment was ingested, processed and egressed, particularly sediment smaller than 10 µm (Turon, Galera & Uriz, 1997; Yahel et al., 2007; Topçu et al., 2010). In order to verify sediment ingestion, future experiments would need to verify intracellular sediment using sectioning and transmission electron microscopy (TEM).

Sponges exposed to acute sediment exposures closed their oscula within 24 h, indicating a cessation of pumping. Internal sediments could have entered the aquiferous system before full oscula closure. To our knowledge, this is the first report of oscula closure in this species, and the first report of closure in response to sediment exposure. A preliminary experiment found no sediment inside dead and anesthetised sponges, indicating active pumping by the sponge is necessary for sediments to enter the sponges. Therefore, osculum closure is a clear response to limit sediment uptake. This closure may even impose a threshold of maximum sediment uptake, which would explain why there was no significant difference in internal sediment concentrations between the high and medium treatments in the acute exposure experiment after 48 h of sediment exposure and 1 h of recovery. Oscula closure leading to cessation of pumping has been observed in a number of other sponge species (e.g., Reiswig, 1971), particularly in response to acute and chronic SSC exposures (Strehlow et al., 2016a; Pineda et al., 2017a). With this prolonged closure, tissue regression and necrosis of sponges was significantly higher in the chronic, highest sediment treatment (45 ± 12%) than in the control treatments (4 ± 1%), and continued to be high throughout the recovery period (30 ± 5%, Pineda et al., 2017b). This regression corresponded to a decrease in the percentage volume of the aquiferous system. Sponge tissue regression results in a dormant state where choanocyte chamber density decreases (Luter, Whalan & Webster, 2012b), the volume of the aquiferous system decreases and, presumably, active pumping all but ceases as there are no longer visible oscula (B Strehlow, pers. obs., 2016). Necrosis, on the other hand, was characterised by a loss of cells which resulted in exposure of the sponge skeletal elements. Tissue regression has also been observed in sponges undergoing physiologically stressful events such as spawning (Abdul Wahab et al., 2016), yet *I. basta* has a remarkable recovery capacity, exhibiting complete recovery within 72 h of being in a fully regressed state (Luter, Whalan & Webster, 2012b).

This dormant state would be detrimental to *I. basta* in the long term as they depend on pumping for vital physiological processes (Bell et al., 2015; Schönberg, 2015; Schönberg, 2016). However, entering into a short-term regressed or dormant state may be beneficial or preferential under chronic sediment pressure. Pumping is energetically costly (Leys et al., 2011), and previous experiments have shown that sponges decrease or cease pumping when exposed to suspended sediments (Gerrodette & Flechsig, 1979; Tompkins-MacDonald & Leys, 2008; Strehlow et al., 2016a). Sponge choanocytes have the fastest cell cycle noted for a multicellular organism, and old cells are constantly expelled (De Goeij et al., 2009). With such a high rate of cellular turnover, maintaining the aquiferous system is likely to be energetically costly. Entering this dormant state would not only decrease the energetic cost of pumping but also the costs of maintaining choanocyte turnover.

## Conclusion

X-ray microscopy proved a useful tool for studying sediment stress and uptake in filter feeders. The resolution (micrometres) that can be achieved over large volumes (millimetres) allowed for sensitive and accurate detection and quantification of introduced particles. The non-destructive nature of the approach allowed sponges to be visualised and quantified without disturbing internal structures or the positioning of introduced particles. Using this tool, we demonstrated that *I. basta* can readily accumulate and expel introduced sediments in short time frames. Coupling X-ray microscopy data with SEM observations led to the identification of five mechanisms that control sediment rejection and elimination in *I. basta* and underpin the tolerance and resilience of this species to sediment related stressors. These mechanism include: (i) mucus production, (ii) exclusion of particles by incurrent pores, (iii) closure of oscula and pumping cessation, (iv) expulsion of particles from the aquiferous system, and (v) tissue regression to reduce the volume of the aquiferous system, limiting pumping and filtration capacity, thereby entering a dormant state. These mechanisms, combined with the compact ultrastructure and relative sediment tolerance of *I. basta*, make it an excellent model species for studying sediment response mechanisms in sponges. Understanding these mechanisms contributes to our general knowledge of the effects of dredging and increased sediment loads on sponges, which will inform effective management of coastal areas given the increasing sediment loads therein.

##  Supplemental Information

10.7717/peerj.3904/supp-1 Figure S1 Raw data for chronic sediment exposures

10.7717/peerj.3904/supp-2 Figure S2 Raw data for acute sediment exposure

10.7717/peerj.3904/supp-3 Movie S1 3D X-ray of *Ianthella basta* after 24 h of acute sediment exposure to siliciclastic sediments in the high sediment (135 ± 30 mg L^−1^) treatment

10.7717/peerj.3904/supp-4 Movie S2 3D X-ray of *Ianthella basta* after 1 h of recovery in control conditions (0 mg L^−1^) after 48 h of acute sediment exposure to siliciclastic sediments in the high sediment (135 ± 30 mg L^−1^) treatment

10.7717/peerj.3904/supp-5 Movie S3 3D X-ray of *Ianthella basta* after 24 h of recovery in control conditions (0 mg L^−1^) after 48 h of acute sediment exposure to siliciclastic sediments in the high sediment (135 ± 30 mg L^−1^) treatment

10.7717/peerj.3904/supp-6 Movie S4 3D X-ray of *Ianthella basta* after 3 d of recovery in control conditions (0 mg L^−1^) after 48 h of acute sediment exposure to siliciclastic sediments in the high sediment (135 ± 30 mg L^−1^) treatment

10.7717/peerj.3904/supp-7 Movie S5 3D X-ray of *Ianthella basta* after 3 w of recovery in control conditions (0 mg L^−1^) after 48 h of acute sediment exposure to siliciclastic sediments in the high sediment (135 ± 30 mg L^−1^) treatment

## Additional Information and Declarations

### Field Study Permissions

The following information was supplied relating to field study approvals (i.e., approving body and any reference numbers): All collections from the Great Barrier Reef were performed under Great Barrier Reef Marine Park Regulations 1983 (Commonwealth) and Marine Parks regulations 2006 (Queensland).

### Data Availability

The following information was supplied regarding data availability: The raw data for Figures 2 and 6 are included as Supplementary Files. Raw 3D data from X-ray microscopy scans can be accessed at: 10.4225/23/59f7d191d7c0e.
